# The role of the cingulum in deep brain stimulation of the limbic pain matrix

**DOI:** 10.1093/braincomms/fcaf225

**Published:** 2025-06-07

**Authors:** Thomas Kinfe

**Affiliations:** Department of Neurosurgery, Mannheim Center for Neuromodulation and Neuroprosthetics (MCNN), Medical Faculty Mannheim, Heidelberg University, 68167 Mannheim, Germany; Mannheim Center for Translational Neuroscience (MCTN), Medical Faculty Mannheim, Heidelberg University, 68167 Mannheim, Germany; Mannheim Comprehensive Medical Systems Technology Campus (MCSC), Medical Faculty Mannheim, Heidelberg University, 68167 Mannheim, Germany

## Abstract

This scientific commentary refers to ‘The cingulum: a central hotspot for the battle against chronic intractable pain?’, by Kollenburg *et al*. (https://doi.org/10.1093/braincomms/fcae368) and ‘The cingulum: anatomy, connectivity and what goes beyond’, by Kollenburg *et al*. (https://doi.org/10.1093/braincomms/fcaf048).

**This scientific commentary refers to ‘The cingulum: a central hotspot for the battle against chronic intractable pain?’, by Kollenburg *et al*. (https://doi.org/10.1093/braincomms/fcae368) and ‘The cingulum: anatomy, connectivity and what goes beyond’, by Kollenburg *et al*. (https://doi.org/10.1093/braincomms/fcaf048).**

This scientific commentary focuses on two articles by Kollenburg *et al*.^1^ published recently in *Brain Communications*: ‘The cingulum: a central hotspot for the battle against chronic intractable pain?’^1^ and ‘The cingulum: anatomy, connectivity and what goes beyond’.^2^ While the first article provides comparative data for both so-called last resort stereotactic interventional therapies, in particular radiofrequency ablation (RFA) versus deep brain stimulation of the anterior cingulate cortex (ACC-DBS), the second article elaborates on anatomical and functional connectivity of the ACC, in particular brain circuits relevant for pain transmission and processing. In addition, alternative non-invasive neurotherapeutics such as transcranial magnetic stimulation of the ACC are briefly discussed.^1,2^

RFA-ACC has been applied for many decades, initially targeting neuropsychiatric disorders (e.g. depression, anxiety, obsessive-compulsive disorders) and then extended towards the treatment of chronic pain conditions. Human studies indicate that the ACC represents a central hub in the limbic pain matrix which interacts with and modulates the affective domain of chronic pain, in contrast to the sensory thalamic nuclei (VPL-VPM, nucleus ventralis posterolateralis), evoking a change in pain perception and pain behaviour.^3^ The development and maintenance of chronic pain arise as a consequence of a deteriorated interplay between different brain networks (default mode network, salience network, cognitive-executive network). Therefore, ablation of the ACC has been suspected to re-balance the salience network, leading to changes in pain attention.^3^ Nascent developments in the field of neuroimaging have changed the neurosurgical workflow permitting visualisation and direct targeting of the ACC. However, there is a broad variety in terms of dose-response relationship, as in some studies different thermocoagulation electrode diameters are used (4–10 mm), and lesioning protocols that vary in time, temperature and duration, with the consequence of considerable variabilities related to the ablated brain volumes. Kollenburg *et al.*^1^ reported a short-term objective response rate ranging from 43% to 53%, depending on the pain disorder, and found ACC-RFA to be effective after 12 months in 76% to 82% with the necessity of re-ablation in a minor subset of patients. Seizure and intracranial haematoma are described as the most frequent adverse events, along with somatic and psychiatric deterioration (urinary incontinence, akinesia, psychomotor deterioration, affect changes, and autism), the vast majority of which resolved and were temporary in character.^1^

Given the negative results in clinical trials and non-surgical therapeutic options, thalamic DBS approval was refused in the United States in 1991. Contrary to this sceptical attitude, the use of thalamic DBS has steadily increased, supported by DBS hardware (e.g. directional leads) and DBS software developments (advanced programming waveforms), and the integration of functional and structural neuroimaging modalities. Human studies indicate, that despite VPL/VPM and periventricular/peri-aqueductal grey (PVG/PAG), key players of limbic pain circuits including the central-lateral thalamic nuclei pars posterior (CLT), the ventral striatum (VS), the anterior limb of the capsule interna (ALIC) and the ACC have been targeted by DBS surgery. This accounts for other stereotactic interventional therapies since RFA and MR-guided focused ultrasound (HIFU) studies reported a meaningful responsiveness in chronic pain patients supporting the therapeutic relevance of the affective pain pathways.^3-7^

A systematic review which analysed DBS efficacy for the treatment of pain disorders identified relevant differences in 228 DBS patients related to target verification comparing DBS surgery with and without microelectrode recording. Furthermore, the necessity and predictive value of DBS trial stimulation are discussed as DBS trial failures were reported in 2% to 48% of implanted patients. In most of the reported cases, DBS leads were inserted either in the VPL/VPM alone or combined with PAG/PVG. Notably, immediate (short-term) DBS response was observed in PAG/PVG and VPL/VPM stimulation, while reduction in pain perception occurred after latency when DBS approached the CmPF, the VS/ALIC and the ACC.^3^

VPL-DBS surgery usually is guided by the clinical intra-procedural response based on the distinct anatomical distribution of body parts within the VPL (head, upper/lower extremities) and promotes paraesthesia in the stimulated pain region of the body within DBS lead implantation. This does not account for the CmPf, the CLT, the VS/ALIC and the ACC, which lack these segregated neuroanatomical borders representative for different parts of the body and being associated with the affective-related pain sphere of pain. Chronic pain patients treated with adjunctive CmPf-DBS or ACC-DBS predominantly reported changes in attentional and emotional pain perception, otherwise described as affective distancing to the chronic pain stimuli. The bidirectional relationships of the ACC with the CmPf (predominantly), the VPL/VPM, the amygdala, the insular, the VS/ALIC and the dorsolateral prefrontal cortex (dlPFC) are crucial for the aversive processing of sensory and nociceptive inputs. Additionally to the CmPf and the ACC, the VS/ALIC has emerged as novel DBS target for pain as the VS/ALIC operates at the crossroad of fronto-striatal circuits, thus linking the orbitofrontal cortex with thalamocortical signalling relevant for anxiety and reward.^8^ Given these facts, Kollenburg *et al*.^1^ are commended for their efforts and work not only reporting ACC DBS outcome, but providing a distinct neuroanatomical frame and explaining the effects observed in clinical use of ACC DBS.^1-3^

Under a rigorous study protocol (randomized-sham controlled cross-over design) VS/ALIC DBS was assessed in a small-scale trial (9 post-stroke pain patients) under a cross-over sham versus verum stimulation umbrella, followed by an open label phase covering 18 months. This study protocol derived from observations in psychiatric patients suffering from obsessive-compulsive disorder and treatment-resistant depression and indicates the crucial function of the VS and the ALIC based on their projections from orbitofrontal to striatal-thalamic-cortical areas, thus impacting mood regulation and interfering with pain attention and anticipation. While primary and secondary endpoints defined as 50% pain reduction failed in that study, an improvement of emotion-related measures was observed. Similar to ACC DBS, seizures, cognitive impairment and dysexecutive deteriorations were recorded. DBS outcome was determined under on/off conditions in a subset of these post-stroke pain patients with active DBS decreasing heat-stimuli evoked activation of the thalamus, insula, operculum and the orbitofrontal area compared with age-/gender matched healthy controls. Furthermore, MEG measures demonstrated that DBS ‘ON’ significantly impacted event-related tasks compared with baseline and deactivated DBS.^6,7^

Nociceptive fibres (tractus spinothalamicus) project via the VPL and VPM to the primary and secondary somatosensory cortex classified as a domain of the sensory pain matrix, which permits conscious and discriminative perception of pain. In contrast to the CmPf and the ACC, the VPL displays a somatotopic organisation and distribution. This distinct functional-structural border within the VPL receives afferent projections from the lemniscus system, the globus pallidus and the cerebellar nuclei and are in turn connected the prefrontal cortex, orbitofrontal cortex and posterior parietal lobe (Fig. 1). In parallel, these nociceptive projections traffic to the intra-laminar thalamic nuclei and the CmPf with the Cm-complex of the CmPf being linked with the internal globus pallidus and the Pf-complex with the brainstem. These intra-laminar thalamic nuclei modulate the dlPFC, the ACC, the entorhinal cortex and the parieto-temporal area, permitting the ACC to get access to descending pain inhibiting tracts of the brainstem and associated spinal cord circuits (Fig. 1).^3^

**Figure 1:**
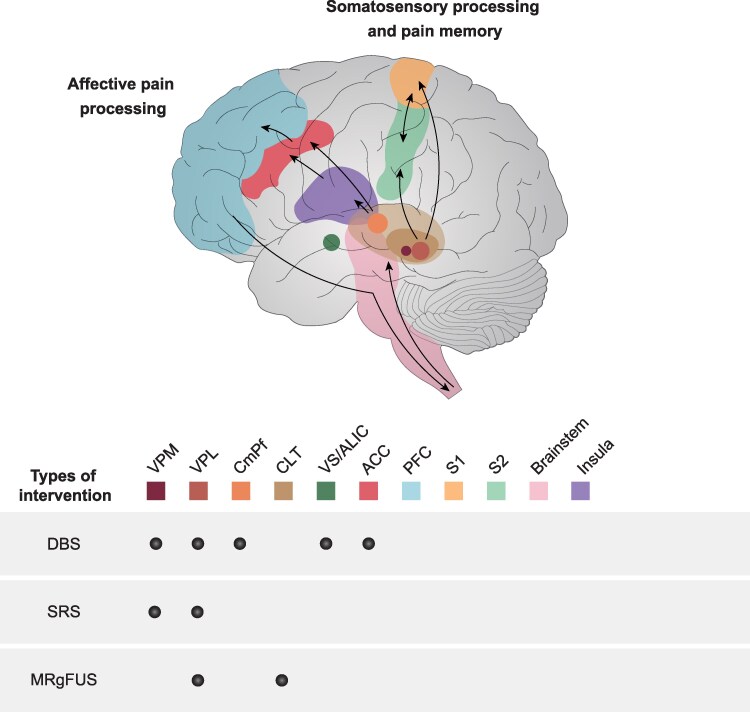
**Projections of the sensory and affective pain circuits relevant for stereotactic interventions.** Displayed are the ascending fibres and projections to the thalamic nuclei stemming from the brainstem and spinal cord, relevant for neural transmission of somatosensory inputs and pain memory signalling. The sensory thalamic VPL is reciprocally connected with the somatosensory cortex with distinct distribution and interaction between the primary (S1) and secondary (S2) areas. Emotional pain regulation and pain attention is encoded within a circuit encompassing the CmPF, which in turn signals to the insula, the ACC, the PFC, the brainstem and descending fibres of the spinal cord. Stereotactic procedures (DBS; SRS; MRgFUS) approaching different targets (CmPf, ACC, VS/ALIC, CLT) for the treatment of chronic pain. Adapted from Nüssel *et al*.^3^ Deep Brain Stimulation, Stereotactic Radiosurgery and High-Intensity Focused Ultrasound Targeting the Limbic Pain Matrix: A Comprehensive Review. *Pain Ther.* 2022;11(2):459–476.

Although not discussed in detail by Kollenburg *et al*.^2^ stereotactic non-invasive, ablative procedures such as MR-guided focussed ultrasound (MRgFUS) and stereotactic radiosurgery (SRS) have targeted the affective pain pathways mainly approaching medial nuclei of the thalamus.^3,9,10^

As for DBS and RFA, the limbic pain circuits have gained increased attention using MRgFUS and SRS. In past years, observational human studies have assessed the efficacy of targeting medial thalamic nuclei (CM, CLT) which reciprocally project to the cingulate cortex. MRgFUS operates under real-time MR thermometry mapping and has received FDA approval and CE mark for the treatment of unilateral movement disorders as well as for neuropathic pain (NP). Incisionless, implant-free MRgFUS uses 650 kHz ultrasonic waves to create thermal irreversible lesions precisely at temperatures higher than 52°C with the patients being awake (fixed in a stereotactic frame). In this way, MRgFUS permits intra-procedural assessment of therapeutic effects if operating at temperature ranges from 40 to 45°C (reversible), therefore potentially limiting MRgFUS-associated adverse events. However, its suitability for the ACC, the CmPF and the CLT remains largely limited (Fig. 1).^3,9,10^

Gallay *et al*.^10^ targeted the CLT using MRgFUS in 12 NP patients and induced lesions within an accuracy comparable to DBS (diameter 3 mm/deviation of 1 mm). Conclusively, a mean pain reduction of 49% after 3 months post-lesioning was determined in 9 patients along with 57% pain reduction after 1 year in 8 patients. Temporary adverse events associated with MRgFUS treatment included dizziness, headache, nausea and vomiting. Post-MRgFUS neuroimaging demonstrated that in 7 out of 18 lesions were in the CLT, whereas in the remaining 11 subjects, the lesions reached neighbouring nuclei like the pulvinar and the CmPf complex.^10^

However, while the aforementioned stereotactic interventions have made remarkable progress, they still struggle with target prediction (sensory versus affective modulation of pain), as no reliable biomarkers exist for brain modulation in chronic pain, thus providing an effective form of target guidance.
